# Conditional deletion of epithelial IKKβ impairs alveolar formation through apoptosis and decreased VEGF expression during early mouse lung morphogenesis

**DOI:** 10.1186/1465-9921-12-134

**Published:** 2011-10-10

**Authors:** Vedang A Londhe, Tiffany M Maisonet, Benjamin Lopez, Jade-Ming Jeng, Jing Xiao, Changgong Li, Parviz Minoo

**Affiliations:** 1 Department of Pediatrics, Division of Neonatology and Developmental Biology, David Geffen School of Medicine at UCLA, 10833 Le Conte Ave., Mailcode 175217, Los Angeles, CA, USA; 2 Department of Pediatrics, Division of Neonatology, USC Keck School of Medicine, 1801 E. Marengo St., Rm. 1G1, Los Angeles, CA, USA; 3 College of Stomatology, Dalian Medical University, Dalian 116027, China

**Keywords:** Inhibitor of kappa-B kinase beta (IKKβ), alveolar development, alveolar maturation, Nuclear factor κB (NF-κB), Nkx2.1, surfactant protein C (SP-C), thyroid transcription factor (TTF-1), apoptosis, vascular endothelial growth factor (VEGF)

## Abstract

**Background:**

Alveolar septation marks the beginning of the transition from the saccular to alveolar stage of lung development. Inflammation can disrupt this process and permanently impair alveolar formation resulting in alveolar hypoplasia as seen in bronchopulmonary dysplasia in preterm newborns. NF-κB is a transcription factor central to multiple inflammatory and developmental pathways including dorsal-ventral patterning in fruit flies; limb, mammary and submandibular gland development in mice; and branching morphogenesis in chick lungs. We have previously shown that epithelial overexpression of NF-κB accelerates lung maturity using transgenic mice. The purpose of this study was to test our hypothesis that targeted deletion of NF-κB signaling in lung epithelium would impair alveolar formation.

**Methods:**

We generated double transgenic mice with lung epithelium-specific deletion of IKKβ, a known activating kinase upstream of NF-κB, using a cre-*loxP *transgenic recombination strategy. Lungs of resulting progeny were analyzed at embryonic and early postnatal stages to determine specific effects on lung histology, and mRNA and protein expression of relevant lung morphoreulatory genes. Lastly, results measuring expression of the angiogenic factor, VEGF, were confirmed *in vitro *using a siRNA-knockdown strategy in cultured mouse lung epithelial cells.

**Results:**

Our results showed that IKKβ deletion in the lung epithelium transiently decreased alveolar type I and type II cells and myofibroblasts and delayed alveolar formation. These effects were mediated through increased alveolar type II cell apoptosis and decreased epithelial VEGF expression.

**Conclusions:**

These results suggest that epithelial NF-κB plays a critical role in early alveolar development possibly through regulation of VEGF.

## Background

Lung morphogenesis is broadly divided into defined stages that extend from prenatal into early postnatal life including embryonic, pseudoglandular, canalicular, saccular, and alveolar phases. Alveolar formation is a tightly regulated developmental process describing the transition of lung architecture from the saccular to alveolar phenotype that begins with the formation of secondary crests or 'septation' of terminal saccules. Extension of septae is accompanied by thinning via loss of interstitial mesenchymal cells, capillary remodeling and differentiation of cuboidal epithelial cells into surfactant-producing alveolar type II (AT2) cells. Alveolar development is finally completed following an isotropic growth phase during which a portion of alveolar type II cells undergo apoptosis while others differentiate into alveolar type I (AT1) cells as gas-exchange surface area increases to maximal levels [reviewed in [1]]. Although the precise details driving septation, apoptosis, and differentiation of cuboidal cells into alveolar type II cells are not completely understood, numerous factors including transcription factors, signaling molecules, and extracellular matrix components are known to participate in this complex process.

Nuclear Factor kB (NF-κB) is a family of transcription factors involved in regulation of growth, differentiation, and apoptosis of several tissues including embryonic limb, liver, skin, bone, and lung [2-13]. NF-κB exists in the cytoplasm in unstimulated cells as a homo- or heterodimer of five structurally related proteins (RelA (p65), c-Rel, Rel-B, NF-κB1 and NF-κB2) with a conserved Rel-homology domain [14]. Extracellular stimuli such as growth factors, cytokines, and other pathogens activate a cascade of enzymatic reactions acting through inhibitor of IκB-kinases (IKKβ--canonical pathway; IKKα--non-canonical pathway) that lead to release of NF-κB for nuclear translocation and gene transcription. We have previously shown that overexpression of the RelA subunit of NF-κB targeted to lung epithelium increased alveolar type II cells through inhibition of apoptosis [15], confirming previous reports supporting a role of NF-κB in lung morphogenesis [10,16,17].

To date, numerous studies have established the contribution of tissue remodeling through apoptosis as a physiologically relevant event during postnatal alveolar development. While apoptosis in the pseudoglandular and canalicular stages of lung development primarily involves the lung mesenchyme, epithelial apoptosis begins in the canalicular stage and extends through the saccular stage until the completion of alveolar formation [18-20]. Studies have also shown that excessive or premature alveolar epithelial apoptosis may be a central event in the pathogenesis of disorders of alveolar hypoplasia such as BPD [21]. The primary epithelial cell type that undergoes apoptosis during normal lung development is the alveolar type II cell. The fate of AT2 cells may be critically important since they serve as the putative stem cell for AT1 cells responsible for gas exchange, and since they express an abundance of vascular endothelial growth factor (VEGF) [22] critical for pulmonary capillary development. Given the major regulatory function of NF-κB as a controller of apoptosis, this property suggests a potential link between NF-κB signaling and airspace remodeling during alveolar formation.

The purpose of the current study was to investigate the role of NF-κB in regulating lung alveolar development. Based on our previous report that targeted epithelial overexpression of NF-κB induced lung maturation [15], we hypothesized that the converse, namely inactivation of canonical NF-κB signaling through conditional deletion of epithelial IKKβ upstream of NF-κB, would impair alveolar formation. To test this hypothesis, we crossed mice expressing the enzyme Cre recombinase in lung epithelium (*Nkx2.1^Cre^*) to mice containing loxP sites flanking exon 3 of IKKβ (*IKKβ*^F/F^) to generate double transgenic mice (*Nkx2.1^Cre^*; *IKKβ*^F/F^) with deleted IKKβ in lung epithelium. We found that targeted epithelial deletion of IKKβ delayed alveolar formation as demonstrated by fewer alveolar type I and type II cells during early alveolar development. The decrease in epithelial cell numbers was associated with increased cell apoptosis and decreased VEGF expression. Our animal model suggests that baseline expression of epithelial NF-κB plays a critical role in regulating early alveolar development.

## Methods

### Generation of Nkx2.1;IKKß^F/F ^double transgenic mice

Inactivation of NF-κB signaling in lung epithelium by selectively targeting the gene encoding IKKβ was carried out by using a Cre/*loxP *recombination strategy. IKKβ^F/F ^mice (C57BL background strain) were a generous gift of Michael Karin (UCSD) and are homozygous for alleles containing *loxP *sites flanking Exon 3 of the mouse IKKβ gene which codes for the ATP binding site of the IKKβ kinase domain [23,24]. To delete the 'floxed' IKKβ alleles in lung epithelium, IKKβ^F/F ^mice were crossed with transgenic Nkx2.1-Cre mice (kindly provided by S. Anderson, Cornell University, NY). Nkx2.1 promoter activity is localized to the thyroid, forebrain, and lung epithelium during early lung morphogenesis and is subsequently localized in the lung to alveolar type II cells following lung maturation. Our previous experience with this mouse line identifies recombination events throughout the developing lung epithelium using β-galactosidase staining in a Rosa26R reporter mouse line [25,26].

Transgenic mice were identified by PCR genotyping analysis of tail biopsies as previously described [25,27]. PCR primers and conditions were as follows: Nkx2.1-Cre genotyping F: [5'- ACG-AGT-GAT-GAG-GTT-CGC-AA-3']; R: [5'-AGC-GTT-TTC-GTT-CTG-CCA-AT-3']; IKKβ F: [5'-TGA-CCCGGG-AAT-GAA-TAG-CA-3'] and R: [5'-GTC-TTC-AAC-CTC-CCA-AGC-CTT-3']; conditions [2 min denaturation at 95°C followed by 35 cycles of 95/55/72°C for 30 s/30 s/1 min and final extension of 72°C for 5 min].

### Analysis of IKKß allele deletion

Tissue specific deletion of IKKβ was confirmed by PCR of genomic DNA obtained from lung tissue using the following primers for recombination analysis: Primer a: [5'-TGA-CCC-GGG-AAT-GAA-TAG-CA-3']; Primer b: [5'-GTC-TTC-AAC-CTC-CCA-AGC-CTT-3']; Primer c: [5' TAG-TCC-AAC-TGG-CAG-CGA-ATA-C-3']; Primer d: [5'-CGC-CTA-GGT-AAG-ATG-GCT-GTC-T-3'] Primer combination a-b was used to detect the intact floxed allele and c-d was used to detect the allele with Exon 3 deletion following PCR conditions noted above. Deletion of IKKβ in epithelial cells was further verified by quantification of IKKβ mRNA and protein in lung tissue. Real-time quantitative PCR analysis of lung mRNA from total lung RNA extraction using primers specific for murine IKKβ (Ikbkb) was performed as described below to compare IKKβ levels in double transgenic mice to littermate controls (see mRNA analysis below). Deletion of IKKß protein in epithelial cells was demonstrated via immunohistochemical analysis using an antibody specific for murine IKKβ (Abcam Inc., Cambridge, MA) (see Immunohistochemistry below).

### Animal Husbandry

All mice were maintained in a barrier facility, and animals were handled under IACUC approved protocols. No serological evidence of viral or bacterial pathogens was detected in sentinel mice maintained with the colony and no evidence of infection was noted at necropsy. The Nkx2.1-Cre mice were maintained and bred as hemizygous and the IKKβ^F/F ^as homozygous colonies.

### Histology and Immunohistochemistry

Tissue sections were prepared from select fetal (E12-E18), neonatal (postnatal days 0 & 7), and young adulthood (postnatal day 30) mouse lungs fixed in 4% Paraformaldehyde solution. To obtain fetal tissue, dams were euthanized using intraperitoneal pentobarbital (100 mg/kg) and fetuses were removed by hysterotomy. Embryonic lungs were then carefully dissected en-bloc and immersed in fixative overnight. Postnatal animals were euthanized as above and the thoracic cavity was then exposed and lungs were perfused free of blood with 1 ml 0.9% normal saline via the spontaneously beating right ventricle under constant pressure of 25 cm H_2_O. A 26 gauge angiocatheter was used to cannulate the trachea and inflation-fix the lungs at 25 cm H_2_O for 1 min. Tissue was immersion-fixed overnight, dehydrated through a series of alcohols and CitriSolv^® ^solution (Fisher Scientific, Pittsburgh, PA), and embedded in paraffin. Serial 5-μm-thick sections were cut and placed on glass slides. Tissue sections were stained with hematoxylin and eosin in order to analyze histopathological changes in the tissue. In preparation for immunohistochemistry, slides were cleared in CitriSolv^® ^and rehydrated through a graded series of alcohols. Slides were then stained using the Histomouse Streptavidin Peroxidase kit (Zymed Laboratories Inc., South San Francisco, CA) as described by the manufacturer. The optimal dilution for the rabbit anti-ki67 antibody (Cell Signaling Technology, Inc., Danvers, MA) was 1:50 dilution, rabbit anti-TTF-1 antibody (Seven Hills Bioreagents, Cincinnati, OH) was 1:1000 dilution, rabbit anti-IKKβ antibody (Abcam Inc., Cambridge, MA) was 1:50 dilution, rabbit anti-Pro SP-C antibody (Seven Hills Bioreagents, Cincinnati, OH) was 1:1000 dilution, mouse monoclonal anti-αSMA antibody (Sigma, Inc., St. Louis, MO) was 1:500 dilution, and goat anti-VEGF antibody (Santa Cruz Biotechnology, Inc., Santa Cruz, CA) was 1:50 dilution. Normal rabbit serum or mouse IgG was used in place of the primary antibody in negative control slides as appropriate. The T1α antibody developed by Dr. Andrew Farr was used at 1:100 dilution and was obtained from the Developmental Studies Hybridoma Bank developed under the auspices of the NICHD and maintained by The University of Iowa, Department of Biological Sciences, Iowa City, IA 52242. A fluorescein anti-hamster or anti-mouse IgG secondary antibody (Vector Laboratories, Inc., Burlingame, CA) was also used at 1:100 dilution (15 μg/ml) as suggested by the manufacturer. All negative control slides showed no immunostain.

### Lung Morphometric Analysis

Four random 5 μm paraffin-embedded tissue sections were taken from three different double transgenic Nkx2.1; IKKβ^F/F ^and control IKKβ^F/F ^lungs at specified stages and separately stained with hematoxylin and eosin or immunostained with anti-TTF-1 or anti-SP-C antibody as described above. The sections were photographed using a SPOT Insight QE camera and software (Diagnostic Instruments, Inc., Sterling Heights, MI) and each image file was analyzed in a blinded manner using the NIH ImageJ 1.37 v program to quantify the number of cells stained positively for the specified antibody. The mean ± SEM was generated and compared between double transgenic and control sections.

Radial alveolar counts were measured as previously described [28]. Briefly, a line was drawn from the center of a respiratory bronchiole to the nearest interlobular septum, to which an intercept line was drawn perpendicularly. The number of distal air sacs that were transected by the intercept line was counted. This assessment was repeated for a minimum of ten terminal respiratory units in one random tissue section per mouse. The mean ± SEM was generated and compared between double transgenic and control sections.

Quantification of alveolar type I cells was estimated using the NIH ImageJ 1.37 v program by first converting T1α immunofluorescence images into a binary composite highlighting all pixels specific for the corresponding color channel (Cy3). The total number of pixels was then summated and compared from a minimum of 5 sections for each transgenic and wild type condition as previously described [29].

### Hart's elastin stain

As previously described [30] slides were cleared in CitriSolv^®^, rehydrated through graded alcohols and then incubated overnight in Hart's Solution (9:1 solution of Weigert's Iron resorcin fuchsin and 1% HCl. Slides were then rinsed in ethanol, 1% HCl, and water before being incubated for 1 hour in Weigert's Iron Hematoxylin, rinsed in water, and then incubated for 5 minutes in Van Gieson's Picro-Fuchsin. Slides were dehydrated through graded alcohols, mounted with a xylene based mounting medium, and photographed using a SPOT Insight QE camera and software (Diagnostic Instruments, Inc., Sterling Heights, MI).

### mRNA Analysis

mRNA was extracted from lungs of double transgenic Nkx2.1; IKKβ^F/F ^mice and control IKKβ^F/F ^littermate controls at specified embryonic and postnatal stages by snap-freezing immediately after dissection and transfer to -80°C until homogenization using a hand-held power homogenizer followed by subsequent organic-solvent extraction with TRIzol and chloroform (Invitrogen Corp., Carlsbad, CA) as described by the manufacturer. Total RNA (1 μg) was reverse-transcribed into cDNA and amplified using the TaqMan Reverse Transcription Kit PCR Kit (Applied Biosystems, Foster City, CA).

Real-time quantitative PCR using primers to detect mouse IKKβ (Ikbkb), murine Nkx2.1, also referred to as thyroid transcription factor 1 (Titf1), murine elastin (Eln), murine PDGF-A (Pdgfa), murine alpha smooth muscle actin (Acta2), murine Caspase 3 (Casp3), murine IL-1β (Il1b), murine CXCL1/KC (Cxcl1), murine CXCR2 (Cxcr2), murine VEGF (Vegfa), murine GAPDH (Gapdh), and housekeeping gene mouse ß-actin (ACTB) was performed on a ABI PRISM 7700 Sequence Detection System using Pre-Developed TaqMan Assay Reagents (Applied Biosystems, Foster City, CA). Quantitative analysis of gene expression was performed using the comparative *C*_T _(Δ*C*_T_) method, in which *C*_T _is the threshold cycle number (the minimum number of cycles needed before the product can be detected) [15]. The arithmetic formula for the Δ*C*_T _method is the difference in threshold cycles for a target (i.e. IKKβ) and an endogenous reference (i.e. housekeeping gene β-actin). The amount of target normalized to an endogenous reference (i.e. IKKβ in double transgenic animals) and relative to a calibration normalized to an endogenous reference (i.e. IKKβ in controls) is given by 2^-ΔΔ*C*T^.

### TUNEL assay for apoptosis

Paraffin-embedded tissue sections prepared from select embryonic and postnatal day lungs of double transgenic Nkx2.1;IKKß^F/F ^and control IKKβ^F/F ^mice were processed using a terminal deoxynucleotidyl transferase-mediated dUTP nick end-labeling (TUNEL) detection kit as described by the manufacturer (*In situ *Cell Death Detection Kit, Roche Applied Science, Indianapolis, IN). Briefly, tissue sections were deparaffinized and rehydrated through a graded series of alcohols and washed in distilled-deionized H_2_O. Following treatment with proteinase K, the slides were washed with PBS and incubated at 37°C for 1 h with TUNEL reaction mixture containing fluorescein-tagged dUTP antibody (to detect DNA strand breaks) per kit instructions. Samples were analyzed by fluorescence microscopy using a Zeiss Axioplan microscope and fluorescence detection system (OPTI-QUIP, Inc., Highland Mills, NY) to detect differences in apoptotic cells in transgenic versus wild-type lungs. Data were quantified by performing manual cell count of TUNEL-positive cells for each section.

### siRNA knockdown of IKKβ

MLE-15 (immortalized murine distal respiratory epithelium) cells were cultured at 37°C in a humidified chamber supplemented with 5% CO_2_. Reverse transfection with Silencer Select siRNA (Applied Biosystems, Foster City, CA) was used with Lipofectamine RNAiMAX (Invitrogen Corp., Carlsbad, CA) to interrogate MLE-15 cell response to knockdown of IKKβ, VEGF, and GAPHD according to the manufacturer's protocol. After optimizing transfection of the siRNA, cells were collected and total mRNA was isolated for real time quantitative PCR analysis. GAPDH siRNA transfected cells were used as a positive control and markers for cell death were analyzed for quality assurance. Real-time quantitative PCR using primers to detect mouse IKKβ (Ikbkb), murine Caspase 3 (Casp3), murine Bcl2 (Bcl2), murine VEGF (Vegfa), murine GAPDH (Gapdh), and housekeeping gene mouse ß-actin (ACTB) was performed on a ABI PRISM 7700 Sequence Detection System using Pre-Developed TaqMan Assay Reagents (Applied Biosystems, Foster City, CA) as described above.

### Statistical Analysis

Data were analyzed using the Microsoft Excel 2003 statistical package (Microsoft Corporation). Two group comparisons were evaluated using the unpaired *Student's t *test. Data were expressed as mean ± SEM where appropriate.

## Results

### Global deletion of the IKKα subunit of IκB kinase complex disrupts lung saccularization

**Figure 1:**
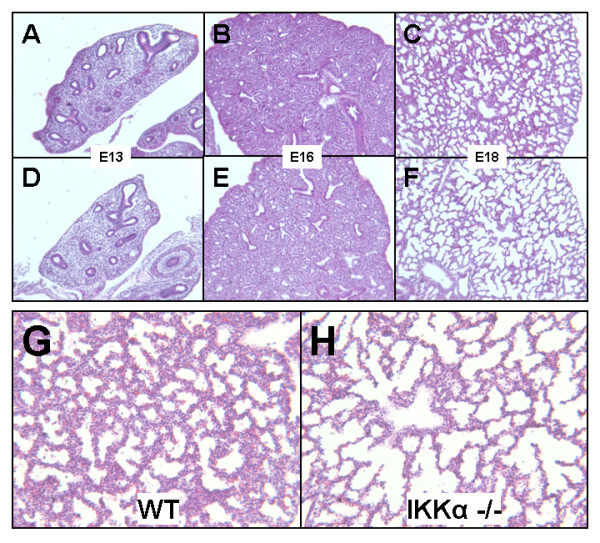
**Global deletion of IKKα disrupts saccular formation**. Representative hematoxylin and eosin stained photomicrographs of lung tissue from embryonic stages E13-E18 from wild type littermate controls (A-C; G) and homozygous IKKα -/- knockout progeny (D-F; H). [4 × magnification in A-F; 10 × magnification in G-H (E18)].

**Figure 2:**
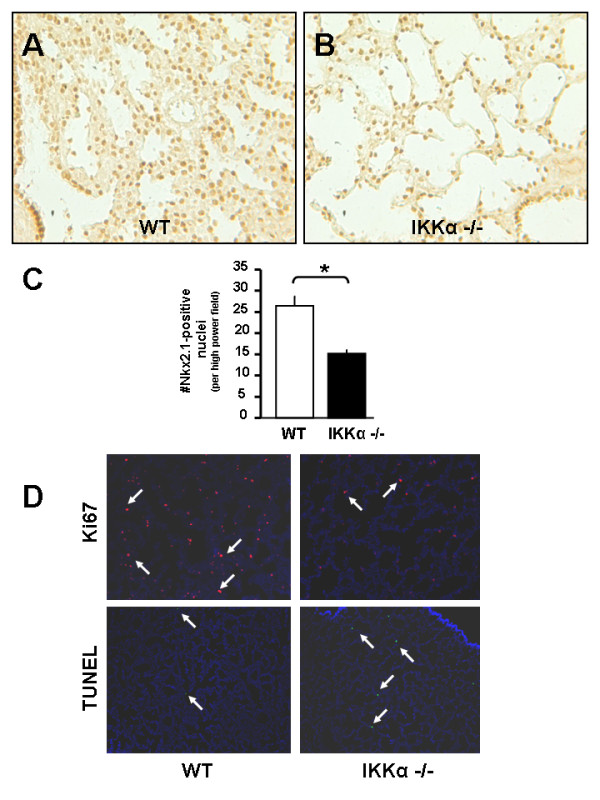
**Global deletion of IKKα decreases alveolar type II cell precursors**. (A, B) Immunohistochemistry for Nkx2.1 with quantification of Nkx2.1-positive cells in (C) at E18. (D) Immunohistochemistry representing proliferation marker ki67 (top panels) with TUNEL stain representing cell apoptosis (bottom panels). [20 × magnification in A-B; 4 × magnification in D; Data are expressed as Mean ± SEM, *p < 0.05 in C].

Since literature describing the developmental impact of key subunit deletions of the IKK complex (IKKα and IKKβ) showed embryonic lethality and did not specifically focus on lung development [8,9,31,32], we determined whether the lungs of IKKα -/- knockout mice exhibit any morphologic changes. A detailed histological analysis of lung sections from IKKα -/- knockouts at E13, E16, and E18 embryonic stages (kindly provided by Inder Verma, UCSD) showed that while no obvious differences were noted at E13 or E16, lung sections at E18 had larger airspaces with thinning of mesenchyme when compared to wild type controls [Figure 1A-H]. Quantitative analysis showed a significant decrease by 45% in the number of alveolar type II cell precursors identified by expression of Nkx2.1 [Figure 2A-C]. Notably, immunohistochemical analysis for proliferating cell marker ki67 demonstrated a significant decrease in proliferating cells throughout the lung mesenchyme and epithelium in IKKα -/- knockout lungs as compared to wild type controls. TUNEL staining also showed more epithelial cells undergoing apoptosis in IKKα -/- knockout lungs [Figure 2D]. These data demonstrate that deletion of IKKα influences lung saccularization and suggest that the IKK signaling complex may regulate lung morphogenesis in late gestation through changes in cell proliferation and apoptosis.

### Conditional deletion of IKKβ in lung epithelium

**Figure 3:**
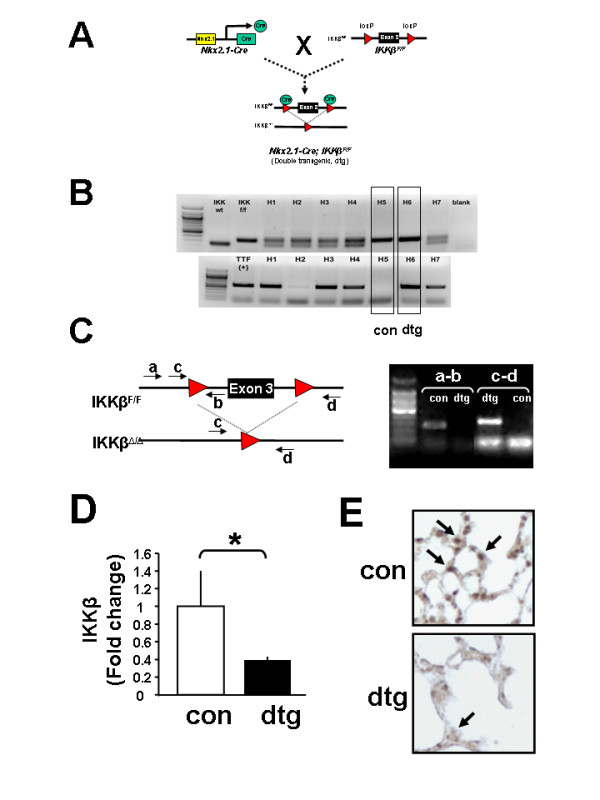
**Generation of Nkx2.1/IKKβ^F/F ^double transgenic mice**. (A) Simplified breeding schema representing cross of Nkx2.1-Cre and homozygous IKKβ^F/F ^mice resulting in deletion of exon 3 of IKKβ gene. (B) Genotyping PCR analysis identifying double transgenic (Nkx2.1/IKKβ^F/F^) and control (IKKβ^F/F^) progeny. (C) PCR primer design to confirm lung epithelium selective deletion of IKKβ. (D) Real-time quantitative PCR and (E) Immunohistochemistry confirming decreased expression of IKKβ in lung tissue. [Data are expressed as Mean ± SEM, *p < 0.05 in D; Arrows pointing to IKKβ-positive alveolar type II cells in top panel and IKKβ-negative alveolar type II cells in bottom panel in (E)].

Given similar reports describing embryonic lethality associated with global deletion of IKKβ [8,31,32], and since IKKβ has been found to be the primary mediator of canonical NF-κB activation and gene transcription [33], we designed a targeted approach for tissue-specific deletion of IKKβ in lung epithelium using a transgenic Cre/loxP recombination strategy. Homozygous floxed IKKβ^F/F ^mice (a kind gift of M. Karin, UCSD) in which Exon 3 of the IKKβ gene is flanked by loxP sites were crossed to mice containing the Nkx2.1-Cre transgene (where Nkx2.1, also referred to as Thyroid transcription factor, TTF-1, is specifically expressed in forebrain, thyroid, and lung epithelium; [25,26]). Following an initial cross to generate heterozygous (Nkx2.1^Cre^; IKKβ^F/+^) animals, these were then back-crossed to homozygous IKKβ^F/F ^mice to generate double-transgenic (Nkx2.1^Cre^; IKKβ^F/F^) progeny [Figure 3A]. Following confirmation of genotype via PCR analysis [Figure 3B], deletion of IKKβ in lung tissue was assessed by PCR analysis using primers designed to detect the presence or deletion of Exon 3 through specific primer combinations [Figure 3C]. Real time quantitative PCR from total lung RNA confirmed a significant decrease (by 60%) in IKKβ mRNA levels in whole lungs of double-transgenic versus control (IKKβ^F/F^) animals [Figure 3D]. Finally, immunohistochemical analysis showed decreased lung IKKβ protein expression in alveolar epithelial cells of double-transgenic lungs [Figure 3E] and a blunted inflammatory response to the endotoxin lipopolysaccharide (LPS) was noted in adult double-transgenic mice as compared to controls (Lopez B, Maisonet TM, Londhe VA: Alveolar NF-κB signaling regulates endotoxin-induced lung inflammation in mice, Submitted). Of note, double-transgenic animals survived normally into adulthood with no obvious signs of sickness or evidence of respiratory compromise. These data demonstrate the successful generation of a mouse model to study the effects of epithelial IKKβ deletion *in vivo*.

### Epithelial deletion of IKKβ delays alveolar formation

**Figure 4:**
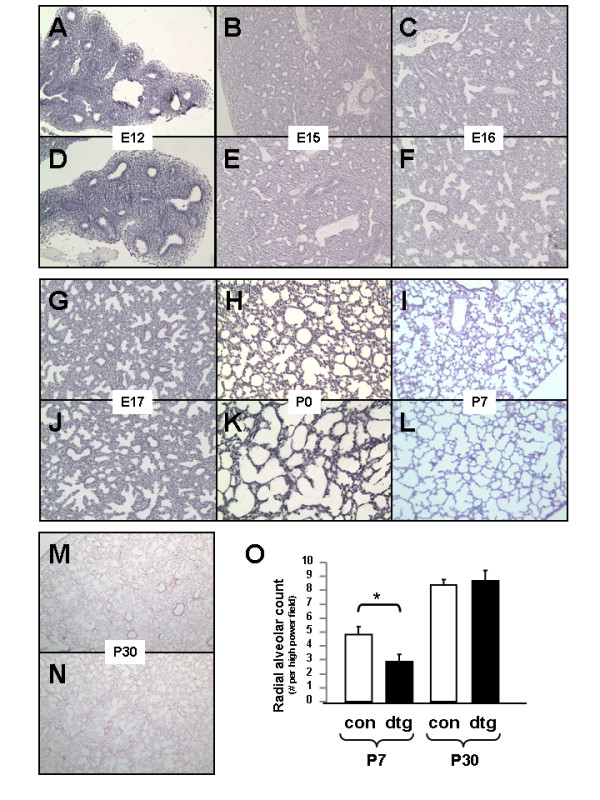
**Epithelial deletion of IKKβ delays alveolar formation**. Representative hematoxylin and eosin stained photomicrographs of lung tissue from embryonic (E12-E17), early postnatal (P0, P7) and young adulthood (P30) stages from IKKβ littermate controls (A-N; top panels) and double transgenic Nkx2.1/IKKβ^F/F ^progeny (A-N; bottom panels). (O) Radial alveolar counts at P7 and P30. [4 × magnification in A-L; Data are expressed as Mean ± SEM, * *p *< 0.05 in O].

Histological analysis of lung sections spanning from E12 thru postnatal stages revealed no differences in lung morphology in embryonic stages until postnatal days P0 and P7, which showed enlargement of saccular and alveolar airspaces in double-transgenic mice [Figure 4A-L]. Alveolar enlargement at P7 was quantified through significant decreases in morphometric radial alveolar counts that correspond to larger alveoli [Figure 4O]. Interestingly, histological findings resolved spontaneously with normalization of radial alveolar counts by the completion of alveolar formation at P30 and remained normal into young adulthood [Figure 4M-O]. Further, a rigorous analysis of multiple serial histological sections from each double-transgenic lung at each postnatal time point showed no evidence of active or resolving inflammation and no significant difference in expression of inflammatory chemokines interleukin (IL-1β), CXCL1/KC (neutrophil chemoattractant), or CXCR2 (neutrophil chemokine receptor) as compared to wild type littermate controls [see Additional File 1]. These data demonstrate that deletion of IKKβ in alveolar epithelium impairs early alveolar formation independent of inflammation.

### Epithelial deletion of IKKβ results in fewer alveolar type I and type II cells

**Figure 5:**
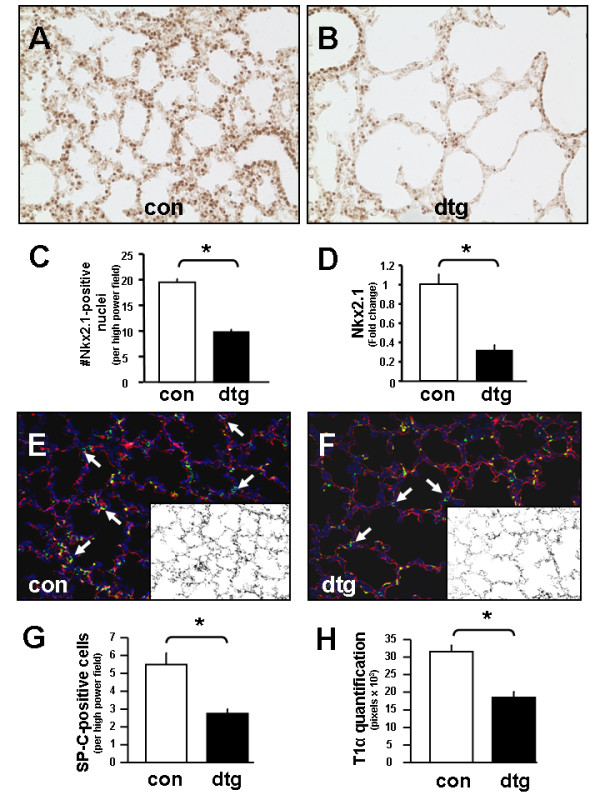
**Epithelial deletion of IKKβ decreases alveolar type I and II cells numbers**. (A, B) Immunohistochemistry for Nkx2.1 with quantification of Nkx2.1-positive cells in (C) at P7. (D) Real-time quantitative PCR confirming decreased expression of Nkx2.1 mRNA in double transgenic compared to control lungs. Immunofluorescence for SP-C (green channel) and T1α (red channel) in control (E) versus double transgenic lungs (F) with respective quantification (G, H) at P7. [10 × magnification; insets represent binary image of T1α-positive pixels; Data are expressed as Mean ± SEM, *p < 0.05 in C-D and G-H].

To determine which specific cell type(s) were affected by epithelial IKKβ deletion, we focused on Nkx2.1 and surfactant protein C (SP-C), known markers for alveolar epithelial cells and precursors to alveolar type II cells. We also examined T1α, expressed by alveolar type I cells, to determine the potential impact on alveolar type II to type I cell differentiation. Immunohistochemical analysis showed a marked decrease in the number of alveolar type II cells in double transgenic lungs compared to controls [Figure 5A-C]. Similarly, real time quantitative PCR also showed decreased Nkx2.1 mRNA levels [Figure 5D]. Quantification of alveolar type I cells was estimated through immunofluorescence for T1α (alveolar type I-specific protein) followed by binary image conversion and summation of pixel numbers corresponding to alveolar type I cell distribution as previously described [29] [Figure 5E &5F]. Results showed proportional decreases in SP-C-positive cells and T1α distribution in double transgenic lungs at P7 [Figure 5G &5H]. These data demonstrate that epithelial IKKβ deletion leads to a proportional decrease in the number of alveolar type I and II cells.

### Alveolar formation is impaired through altered lung septation

**Figure 6:**
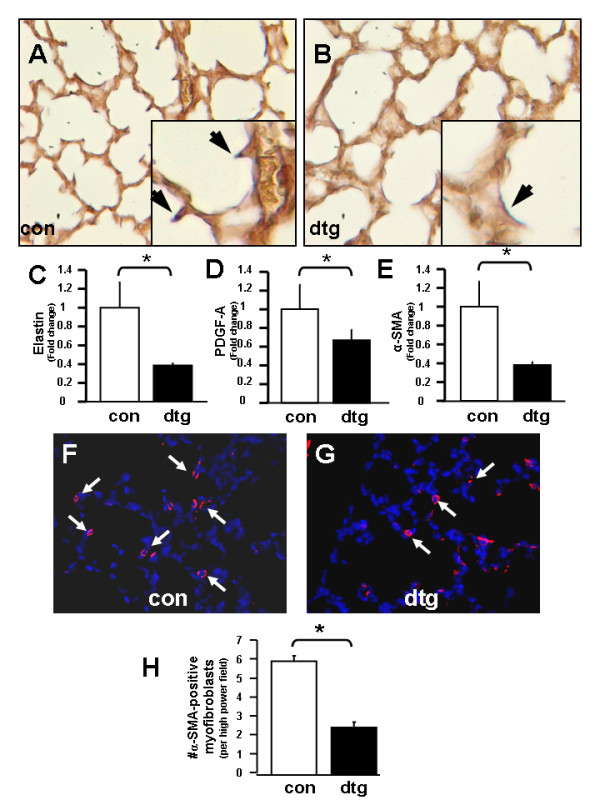
**Alveolar formation is impaired through altered lung septation**. Hart's elastin stain in control (A) versus double transgenic (B) lungs at P7. Real-time quantitative PCR showing expression of (C) elastin, (D) PDGF-A and (E) α-SMA. Immunofluorescence for α-SMA representing myofibroblasts in control (F) versus double transgenic (G) lungs at P7 with quantification in (H). [40 × magnification in A, B, F, G with insets 80 × magnification and arrows pointing to elastin expression in A & B, and arrows pointing to myofibrobasts in F & G; Data are expressed as Mean ± SEM, * *p *< 0.05 in C-E, and H].

To determine which genes may be responsible for the observed alveolar changes in double transgenic lungs, we queried relevant lung morphoregulatory genes known to be expressed at a critical period during alveolar septation. Hart's elastin stain at P7 showed a marked absence of secondary crests expressing elastin, an interstitial matrix protein involved in initiation and progression of alveolar formation [34,35], in double transgenic versus control sections [Figure 6A-B]. Real-time quantitative PCR confirmed a significant decrease in elastin mRNA expression and demonstrated a similar decrease in levels of platelet-derived growth factor (PDGF-A) and α-smooth muscle actin (α-SMA), differentiation markers expressed during alveolar maturation [36] by lung epithelium and lung myofibroblasts, respectively [Figure 6C-E]. In addition, we performed immunofluorescence analysis of α-SMA-positive cells in lung parenchyma to quantify potential changes in myofibroblast numbers. Results showed significant decreases in numbers of myofibroblasts in double transgenic lungs as compared to controls at P7 [Figure 6F-H].

### Epithelial deletion of IKKβ enhances cell apoptosis

**Figure 7:**
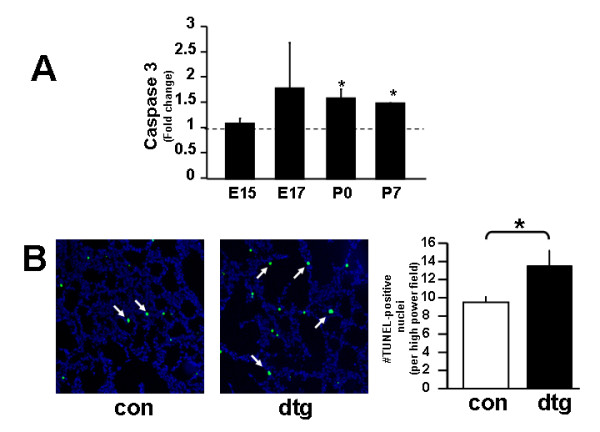
**Epithelial deletion of IKKβ enhances cell apoptosis**. (A) Real-time quantitative PCR showing lung Caspase-3 expression at indicated embryonic and postnatal stages. (B) TUNEL stain with quantification of TUNEL-positive nuclei at P0. [Bars in (A) represent fold induction in double transgenic compared to control lungs; Data are expressed as Mean ± SEM, * *p *< 0.05].

To determine the cellular processes by which epithelial deletion of IKKβ alters secondary crest formation, we analyzed known functions of the IKK/NF-κB signaling complex that include cell proliferation and inhibition of apoptosis [37]. A proliferation assay to detect Ki67, a nuclear protein found in proliferating cells, showed no demonstrable differences between double transgenic versus control lungs (data not shown). In contrast, assays to detect apoptosis via real time quantitative PCR showed that Caspase 3, a marker of apoptosis, was significantly increased in the early postnatal period [Figure 7A]. Similarly, apoptosis detected by TUNEL assay confirmed markedly increased apoptotic cells in an epithelial distribution in double transgenic lungs [Figure 7B]. These data demonstrate that deletion of IKKβ in alveolar epithelium leads to fewer alveolar type II cells through increased cell apoptosis and identifies regulation of cell apoptosis as an important function of NF-κB signaling during alveolar development.

### Epithelial deletion of IKKβ decreases VEGF expression

**Figure 8:**
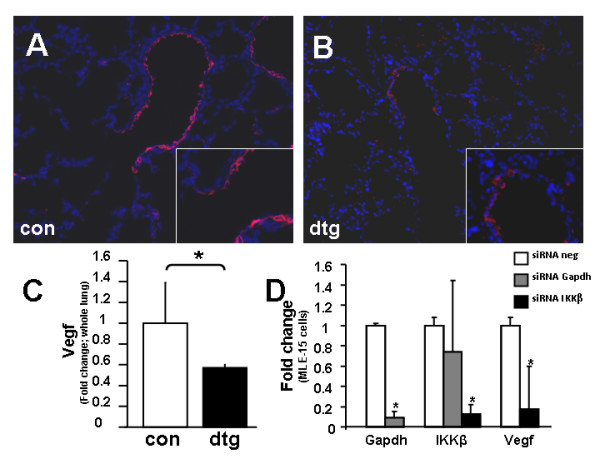
**Epithelial deletion of IKKβ decreases VEGF expression**. Immunofluorescence showing VEGF expression in control (A) versus double transgenic (B) lungs. (C) Real-time quantitative PCR showing VEGF expression. (D) Real-time quantitative PCR from siRNA knockdown in MLE-15 cells *in vitro *showing Gapdh, Vegf, and IKKβ expression. [Data are expressed as Mean ± SEM, *p < 0.05].

Finally, to identify the molecular mechanisms of delayed alveolar formation from loss of AT2 cells in our model, we measured expression of VEGF, known to be secreted by the alveolar epithelium [22] and an important mediator of epithelial-endothelial signaling. Results showed marked decrease in VEGF protein expression in the distal airways of double transgenic lungs (Figure 8A & B) and significant decrease of total lung VEGF mRNA as compared to controls (Figure 8C). To confirm that VEGF decreased in specific response to deletion of IKKβ, an *in vitro *approach using the immortalized mouse lung epithelial cell line MLE-15 showed that siRNA knockdown of IKKβ significantly decreased VEGF (by 80%). Nonspecific effects of siRNA knockdown were ruled out using appropriate positive and negative controls (Figure 8D). Specifically, siRNA against IKKβ did not induce apoptosis in MLE-15 cells as demonstrated by no change in expression of Bcl2 or Caspase-3 (data not shown). These findings identify the importance of IKKβ/NF-κB signaling in regulating VEGF and epithelial-endothelial crosstalk.

## Discussion

Alveolar formation is a complex developmental program during late gestation/early postnatal life in mammals that can be easily perturbed by inflammation. We previously found that overexpression of the RelA subunit of NF-κB accelerates lung maturation as evidenced by increased alveolar epithelial cells [15]. We therefore hypothesized that inactivation of NF-κB would conversely impair alveolar formation. Our results demonstrated that targeted deletion of IKKβ in the lung epithelium reduces the number of alveolar type I and II cells and transiently impairs alveolar secondary crest formation. IKKβ deletion reduced alveolar epithelial cells through increased cell apoptosis and was associated with decreased VEGF expression. Collectively, these findings highlight an important function of NF-κB in controlling early alveolar development.

Since the original description of NF-κB more than twenty years ago [38,39], a number of studies have investigated its role in developmental organogenesis. Relevant early studies in knockout animals for the RelA (p65) subunit described embryonic lethality at E15.5-E16.5 due to massive liver apoptosis [2]. Similarly, global deletion of IKKβ also resulted in lethality at E13.5-E14.5 from liver apoptosis [8,31,32] while deletion of IKKα proved lethal immediately after birth due to epidermal defects [5,9]. Although the homozygous progeny in these studies were noted to have abnormalities of cell apoptosis in specific organs, no obvious signs of developmental lung abnormalities were reported. Evidence supporting a potential role of NF-κB in lung morphogenesis was first demonstrated in studies of the chick embryo in which experimentally-induced perturbations of NF-κB gene expression in lung mesenchyme resulted in abnormal lung branching morphogenesis [10]. The results showed that overexpression of NF-κB targeted to lung mesenchyme via adenoviral vector delivery inhibited branching during early lung morphogenesis. Additional indirect evidence of NF-κB signaling in fetal mouse lungs was also shown through intra-amniotic injection of LPS with activation of Toll-like receptor (TLR4) upstream of NF-κB that increased alveolar type II cells as described above [17,40]. Taken together with our previous report of maturational effects of NF-κB overexpression [15], these studies support a link between NF-κB and lung morphogenesis.

The property of NF-κB as an inhibitor of apoptosis is well described [37]. Apoptosis describes the final step of a cell's normal lifespan and is a distinct form of programmed cell death characterized by loss of cell function and morphological changes. Apoptosis leads to cell death without inflammation and serves an important function throughout alveolar development, especially during septation. Apoptotic changes are first noted in mesenchymal tissue during early development followed by a shift to both mesenchymal and epithelial tissue layers as alveoli form [19]. Interestingly, rodent models have demonstrated that the numbers of alveolar type II cells (the putative precursors of type I cells) are in excess just prior to the completion of alveolar formation [20]. A transgenic mouse model targeting overexpression of the Fas ligand to lung epithelium recently showed that excessive cell apoptosis caused disruption of alveolar development [21]. The activators that lie upstream of NF-κB and regulate anti-apoptotic properties largely include TNF ligands (Tumor Necrosis Factor) and TNFRs (TNF-receptors) [41,42]. Some TNF/TNFRs activate the expression of inhibitor of apoptosis proteins (IAPs). TNFR ligands also transduce cellular responses through TNFR-associated factors, also known as TRAFs. Specifically, TRAF2 and TRAF5 are known to activate NF-κB and ultimately prevent cell apoptosis. Apoptosis of lung epithelial cells thus emerges as an important process in regulating a balance between those cells that are redundant versus those that ultimately contribute to alveolar maturation.

Results of the current study showed that targeted deletion of IKKβ in lung epithelium increased cell apoptosis and led to early postnatal alveolar hypoplasia. Although previously unreported, global deletion of IKKα also resulted in fewer and larger alveoli in E18 lungs. The significance of these observations is noteworthy since IKKα and IKKβ are known to exert their effects through divergent signaling pathways [37]. IKKα is a participant of both canonical as well as non-canonical NF-κB signal transduction. In the canonical pathway, it forms a heterodimer with IKKβ following activation by stimuli such tumor necrosis factor alpha (TNFα), interleukin-1 (IL-1), or lipopolysaccharide (LPS); whereas in the non-canonical pathway, IKKα forms homodimers with itself after activation by NF-κB -inducing kinase (NIK). Activation of IKKα via this non-canonical pathway engages a pro-proliferative function through induction of p52 and cyclin D promoter activity, and also plays an independent role in regulating differentiation of certain cell types. A minor role in anti-apoptotic function has also been described [33]. In comparison, the consequences of IKKβ activation stimulate largely anti-apoptotic, pro-inflammatory, and some pro-proliferative mechanisms. Our data showing a significant decrease in cell proliferation following global IKKα deletion and an increase in cell apoptosis following targeted IKKβ deletion thus support the concept that NF-κB plays a critical role in regulating cell survival during early alveolar development.

The findings from our study showing decreased epithelial VEGF expression following conditional IKKβ deletion and siRNA knockdown associated with delayed alveolar formation are consistent with previous reports that the VEGF/VEGF-receptor system mediates a link between branching morphogenesis, vascular development, and morphogenesis of the pulmonary epithelium [43]. VEGF (specifically isoform VEGF-A) has been shown to be secreted by peripheral epithelial cells at the tips of developing respiratory tubules. Inactivation of VEGF-A in respiratory epithelium results in almost complete absence of pulmonary capillaries and leads to defects in primary septae formation presumably by interruption of a paracrine interaction necessary for coordination of pulmonary and epithelial vascular development [44]. VEGF deficiency was also recently shown in a mouse model of diabetes-induced respiratory distress syndrome and surfactant deficiency demonstrating its critical angiogenic role required for maturation of alveolar epithelium [45]. Similarly, exogenous intratracheal delivery of VEGF prevented fatal respiratory distress in premature mice [46] and stimulation of VEGF production conferred protection against acute lung injury [47]. Interestingly, new evidence suggests that VEGF itself may also possess an anti-apoptotic function in the context of acute lung injury such as hyperoxia exposure and mechanical stretch of alveolar epithelium [47,48]. It is thus intriguing to consider the possibility that increased AT2 cell apoptosis observed in our model might be a consequence of either direct loss of IKKβ/NF-κB activity or an indirect effect through loss of a protective function of VEGF, although the exact details remain unclear.

Potential limitations of the current study that are important to consider are that Nkx2.1 promoter activity is present in the forebrain and thyroid in addition to the lung epithelium. It is thus conceivable that potential effects on respiratory drive *in utero *or postnatally could lead to changes in lung architecture. Similarly, changes in thyroid metabolism could also have potential impact on lung development. Although these possibilities exist, we did not observe any obvious changes in respiratory pattern or metabolic activity (e.g. weight gain or loss) in double transgenic versus control animals.

## Conclusions

In conclusion, we have developed an animal model in which targeted deletion of NF-κB signaling in the lung epithelium results in alveolar hypoplasia. The clinical relevance of our model may be applicable to disorders of alveolar formation such as bronchopulmonary dysplasia in which the primary pathology appears to be an arrest or impairment of alveolar secondary crest formation. Our model may prove particularly useful to study the molecular mechanisms whereby inflammatory insults or other environmental factors such as oxidative stress, etc., might further impact NF-κB to permanently impair normal lung morphogenesis.

## List of abbreviations

α-SMA: Alpha smooth muscle actin; AT2: alveolar type II cell; IKKβ: Inhibitor of kappa-B kinase beta; IL1: Interleukin 1; LPS: Lipopolysaccharide; NF-κB: Nuclear factor κB; NIK: NF-κB-inducing kinase; PDGF: Platelet-derived growth factor; SP-C: Surfactant protein C; TLR4: Toll-like receptor 4; TNFα: Tumor necrosis factor alpha; TTF-1: Thyroid transcription factor; TUNEL: terminal deoxynucleotidyl transferase-mediated dUTP nick end-labeling; VEGF: Vascular endothelial growth factor

## Competing interests

The authors declare that they have no competing interests.

## Authors' contributions

VL designed the study, participated in generation of the double transgenic animals, and drafted the manuscript. TM carried out the proliferation/apoptosis studies and *in vitro *siRNA knockdown experiments. BL performed the lung morphometric and quantitative analyses. JJ participated in generating the double transgenic animals and lung dissections. JX performed the IKKα knockout histology studies. CL participated in study design and coordination. PM participated in conceiving of the study and helped to draft the manuscript. All authors approved the final manuscript.

## Supplementary Material

Additional file 1 **Expression of inflammatory chemokines**. Realtime quantitative PCR showing no significant differences in IL-1, CXCL1/KC, or CXCR2 expression at P0. [Data are expressed as Mean ± SEM] Click here for file
