# Supplementary figures and images for: Conditional deletion of epithelial IKKβ impairs alveolar formation through apoptosis and decreased VEGF expression during early mouse lung morphogenesis
Source: Respir Res. 2011 Oct 10;12(1):134. doi: 10.1186/1465-9921-12-134 (PMC3202236; doi:10.1186/1465-9921-12-134)

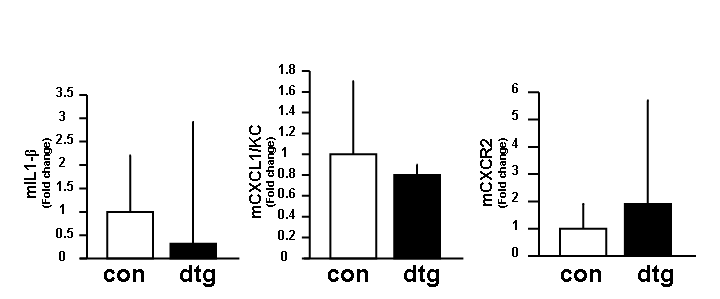

Supplement: Additional file 1 — Expression of inflammatory chemokines. Realtime quantitative PCR showing no significant differences in IL-1, CXCL1/KC, or CXCR2 expression at P0. [Data are expressed as Mean ± SEM] [file 1465-9921-12-134-S1.TIFF]
